# Frequency of Symptoms and Health Seeking Behaviours of Menopausal Women in an Out-Patient Clinic in Port Harcourt, Nigeria

**DOI:** 10.5539/gjhs.v5n4p39

**Published:** 2013-03-18

**Authors:** Paul Owajionyi Dienye, Funsho Judah, Geraldine Ndukwu

**Affiliations:** 1 Department of Family Medicine, University of Port Harcourt Teaching Hospital, Port Harcourt, Nigeria

**Keywords:** menopausal symptoms, prevalence, postmenopausal women, health seeking behaviour, Nigeria

## Abstract

**Objectives:**

This study was carried out to determine the frequency and severity of menopausal symptoms and health seeking behaviour of women with menopausal symptoms attending the General Outpatient Department of the University of Port Harcourt Teaching Hospital.

**Method:**

This is a cross-sectional, descriptive study in which data was collected from menopausal women using a three-part, pre-tested questionnaire for a period of three months (July–September 2010). Part 1 consisted of information regarding socio-demographic and general medical information. Part 2 consisted of the modified version of the menopause rating scale (MRS). Part 3 sought for information on their health seeking behaviour. Data was analysed using EPI INFO version 6.04d software package.

**Results:**

A total of 385 women were recruited with ages ranging from 35 to 95 years, and a mean of 58.4 ± 10.39 years. The most prevalent menopausal symptoms were loss of libido (92.47%), muscle pain (87.53%), joint pain (85.45%) and tiredness (80.26%). Urinary symptoms had the least prevalence (7.79%). Results on the severity of menopausal symptoms showed that 28.25%, 49.84% and 21.9% were experiencing severe, moderate and mild menopausal symptoms, respectively. Loss of libido (79.21 %) was the most severe symptom followed by urinary symptoms (40%). The patent drug dealers were the most consulted (51.4%) followed by health workers (44.7%). The traditional healers were consulted by a small percentage (3.8%).

**Conclusion:**

The most common menopausal symptom among the patients in this study was loss of libido and the least common was urinary symptoms. The symptoms are similar to findings in other parts of the world but their prevalence and severity differ. In spite of the available health facilities in these communities, the utilization of the services of patent drug dealers is still very high but the traditional healers were poorly utilized.

## 1. Introduction

Menopause is an important landmark in the life of a woman (Malik, 2008). It is defined as a physiological event in which there is at least twelve consecutive months of amenorrhoea caused by depletion of ovarian function (Nisar & Sohoo, 2010). This results in various somatic, vasomotor, sexual and psychological symptoms that impair the overall quality of life of women (Nisar et al., 2010; Dennerstein, Dudly, Hopper, Guthrie, & Burger, 2000; Deeks & McCabe, 2004). Menopause can also result from hysterectomy with or without oophorectomy (surgical menopause) and treatment with the gonadotrophic releasing hormone agonist and cytotoxic chemotherapeutic agents (Smoot, 2004).

Menopause has become an important subject of study because of the global increase in life expectancy resulting from better nutrition and improved health care delivery (Lee et al., 2010). The introduction of governmental and other stakeholders’ interventions targeted at achieving the Millennium Development Goals is also expected to increase the population of postmenopausal women. In 1990, the world population of postmenopausal women was reported by Aso (1998) to be 476 million, with 40% living in the industrialized world. It is estimated that this figure will increase to 1200 million with 76% of these women in the developing countries by the year 2030 (Aso, 1998). With the increasing average length of the postmenopausal life span, it has become imperative for healthcare providers to focus more attention on the health of this group of women to ensure that they enjoy this twilight years of their life optimally (Lee et al., 2010). The loss of reproductive capability resulting from menopause is a critical issue that represents the end of fertility and the onset of the aging process.

Symptoms of menopause are multiple and differ from one study to the other. For some women, the menopausal symptoms are severe and disruptive to their activities of daily life but for others they are mild and the transition is acceptable (Cheung, Chaudry, Kapral, Jackevicus, & Robinson, 2004). Robinson (1996) opined that these symptoms may be influenced by a combination of physical changes, cultural influences and individual perceptions and expectations. The variation of symptoms between studies has been attributed to various factors. These studies differ in terms of the specific symptoms studied, the number of symptoms included in the list, the time frame for symptom reporting, the sample characteristics such as age of sample, the composition of sample (some excluded women taking estrogen), or whether the sample was clinic or community based (Im, 2009). In general, menopausal symptoms include both physical and psychological symptoms (Hardy & Kuh, 2002; Li & Holm, 2003; O’Bryant, Palav, & McCaffrey, 2003).

The physical symptoms commonly associated with menopause include weakness, internal heat, waist pains, “false pregnancy”, general body pains, shrinking of the body, dizziness, sweating, restlessness, unhappiness and urinary incontinence. Vaginal symptoms reported include dryness, discomfort, itching and dyspareunia. Sexual problems, particularly loss of libido and primary sleep disorders (apnoea and restless leg syndrome) are widespread in this group of women (Okonofua, Lawal, & Bamgbose, 1990; Robinson, 1996; Grady, 2006). Combination of these factors, such as loss of fertility, fear of ageing with its attendant loss of physical attractiveness, reduced activity and its effect on ones status in the community; and changes in family roles may lead to depression (Cheung et al., 2004). The psychological symptoms include forgetfulness and irritability, insomnia, headache and anxiety. Besides all these effects, menopause can affect the quality of life by being a major cause of morbidity (cardiovascular diseases and osteoporosis) in postmenopausal women (Grodstein et al., 2000; Grodstein, Manson, & Stampfer, 2001).

Menopause is not a disease but the symptoms and their severities which are mainly subjective can be very challenging. Hot flashes, for example, are associated with a decreased quality of life (Groeneveld et al., 1996).

There were no standardized scales for measuring the severity of the symptoms of aging and their impact on the health-related quality of life (HRQOL) until in the early 1990s when the Menopause Rating Scale (MRS) was developed. This is a standardized HRQOL scale with good psychometric characteristics. The severity of menopausal symptoms, as measured using the MRS, is known to clearly reflect the profile of the quality of life (QOL) dimensions. It is therefore useful in the measurement of the QOL of postmenopausal women (Schneider, 2002). Unfortunately many postmenopausal women do not know the health implications of menopause nor coping strategies (Im, 2009). Even some doctors know little about menopause beyond symptoms and details of hormone replacement therapy (Loppie, 2005). An important coping strategy adopted by individuals with medical challenges is to seek for medical attention. The choice of the health provider consulted for a symptom is linked to the perceived cause of the symptom (Ahmed, Sobhan, Islam, & Barkat-e-Khuda, 2001), the severity of the symptoms, socio-cultural influences, distance, place and cost of treatment, income, level of education and quality of health care facilities (Sullivan & O’Conor, 2001).

Few studies have examined menopause symptomatology in primary care settings. To the knowledge of the authors, no such study has been conducted in South-South Nigeria. Most knowledge related to symptoms experienced during the menopausal transition comes from studies of Caucasians (Im, 2009). This leaves a gap in literature since menopause symptoms vary from society to society (Papini, Intrieri, & Goodwin, 2002) because of changes in demographic characteristics, psychosocial and lifestyle factors which are important determinants (Lee et al., 2010). To optimize the health care for middle-aged women, there is need to understand the process by which women describe, explain and experience menopause and also to understand the factors that may shape their experiences. This study was carried out to determine the pattern, severity and health seeking behaviour of women with menopausal symptoms attending the General Outpatient Department of the University of Port Harcourt Teaching Hospital. The findings in this study would form the basis for health promotional activities to make primary care physicians more sensitive to the peculiar needs of postmenopausal women and thereby, improve quality of care.

## 2. Methods

### 2.1 Study Setting

The University of Port Harcourt Teaching Hospital is one of the major tertiary health institutions in the South-South zone of Nigeria. It is the apex health care institution in Port Harcourt, the populous, cosmopolitan capital of Rivers state and epic-centre of the oil and gas industry in the Niger delta region.

### 2.2 Study Population

The study population was made up of postmenopausal women with at least one year of amenorrhoea, not due to surgery or other obvious cause such as severe illness, extreme weight loss and endocrine disorders.

### 2.3 Sample Size Determination

Using the simple statistical formula: n=z^2^pq/d^2^, a sample size of 384 subjects was calculated at a margin of error of 5% and a confidence level of 95%. All eligible and consenting menopausal women who attended the clinic during the period of study were consecutively recruited until the sample size was attained.

### 2.4 Data Collection

Data were collected using a three-part, pre-tested questionnaire, which was administered by the researchers. Part 1 consisted of information regarding socio-demographic and general medical information. Socio-economic class was measured using a simple scoring system developed by Oyedeji (1985) that is based on the level of education of the women. Social Class 1 was awarded to University graduate or its equivalents;

Social Class II to secondary school certificate holders who also had teaching or other professional training;

Social Class III to school certificate or grade II teachers’ certificate holders or its equivalents;

Social Class 1V to those with Junior Secondary School 3 or primary six certificates and

Social Class V to those who could neither read nor write or were illiterates.

Part 2 of the questionnaire consisted of the menopause rating scale (MRS) developed by Heinemann, DoMinh, Strelow, Gerbsch, Schnitker, and Schneider (2006). This was modified for the purpose of this study and used in the assessment of menopausal symptoms and their severity. It comprises of 13 items assessing menopausal symptoms, which could be divided into three subscales. A) Somatic: Hot flushes, heart discomfort, sleep problem and muscles and joint problems. B) Psychological: depression, irritability, anxiety and physical and mental exhaustion. C) Urogenital: Sexual problems, bladder problems and dryness of vagina. Each item can be graded from 0-3, (0= not present), (1=mild), (2=moderate) (3=severe in which case the symptoms were so bothersome that the usual daily activities could not be performed).

Part 3 sought for information on their health seeking behaviour. This part of the study was conducted with a structured questionnaire containing straight dichotomous and close-ended questions. This was designed after informal conversations with menopausal women in the clinic about their health seeking behaviour. To standardize the questionnaire, it was pre-tested on forty menopausal women in the General Out Patient Department of the University of Port Harcourt Teaching Hospital and all ambiguities were corrected. The question asked was, what are your sources of treatment prior to hospital visit? The options included (a) Religious leaders (b) Patent drug dealers (c) Health workers (d) traditional healers (e) Nothing (Answer “Yes” or “No” for each option).

### 2.5 Statistical Analysis

Data collected from the study were recorded in the Microsoft Office Excel 2003 and subsequently analyzed using EPI INFO version 6.04d software package. Approval from the Ethics Committee of the University of Port Harcourt Teaching Hospital was obtained. Informed written consent was received from the women prior to recruitment into the study.

## 3. Results

A total of 385 women were recruited with ages ranging from 35 to 95 years, a mean of 58.4 ± 10.39 years and median age of 58 years. Majority of the participants (36.62%) were within the 45-54 years age group. Patients aged over 95 years constituted the least (0.8%) of the population (Table 1). The level of educational attainment was generally low among the subjects with majority, 151 (39.2%) having no formal education and only 46 (11.9%) attaining tertiary level of education. The most prevalent occupational groups among them were trading (27.3%) and farming (23.4%). Approximately equal numbers of married women, 179 (46.5%) and widows, 178 (46.2%), were represented in the subjects. Only 9 (2.0%) of the women were single, while 19 (4.9%) were divorcees. Most of the subjects belonged to socio-economic classes 4 (44.94%) and 5 (24.94%). The mean age of menopause was 48.3 ± 7.64 years (Table 1).

**Table 1:** Socio-demographic characteristics of the subjects (n=385)

Characteristics	Number (%)
**Age groups (yrs)**	
35-44	14 (3.63)
45-54	141 (36.62)
55-64	129 (33.51)
55-64	129 (33.51)
65-74	68 (17.66)
75-84	23 (5.97)
85-94	7 (1.82)
>95	3 (0.78)

**Educational level**
Non formal	151 (38.96)
Primary	110 (28.6)
Secondary	78 (20.3)
Tertiary	46 (11.9)

**Occupation**
Business	37 (9.6)
Civil Service	36 (9.4)
Farming	90 (23.4)
Housewife	62 (16.1)
Teaching	33 (8.6)
Trading	105 (27.3)
Others	22 (5.7)

**Marital Status**
Single	9 (2.0)
Married	179 (46.5)
Widowed	178 (46.2)
Divorced	19 (4.9)

**Socioeconomic Class**
Class 1	4 (1.04)
Class 2	35 (9.10)
Class 3	77 (20.00)
Class 4	173 (44.94)
Class 5	96 (24.94)

The most prevalent menopausal symptom among the subjects was loss of libido presented by 356 (92.47%) women. This was followed by muscle pain (87.53%), joint pain (85.45%) and tiredness (80.26%) in descending order of prevalence. Urinary symptoms recorded the least prevalence with only 30 participants (7.79%) presenting with such complaint (Table 2). Results on the severity of menopausal symptoms showed that 28.25%, 49.84% and 21.9% were experiencing severe, moderate and mild menopausal symptoms, respectively.

**Table 2:** Prevalence and severity of menopausal symptoms among subjects

Menopausal symptoms	Severity			

	Mild [n(%)]	Moderate [n(%)]	Severe [n(%)]	Total [n(%)]
Loss of libido	31(8.71)	43(12.08)	282(79.21)	356(92.47)
Muscle pains	46(13.65)	237(70.33)	54(16.02)	337(87.53)
Joint pains	57(17.32)	230(69.91)	42(12.77)	329(85.45)
Tiredness	25(8.1)	244(78.96)	40(12.94)	309(80.26)
Hot flushes	62(20.88)	200(67.34)	35(11.78)	297(77.14)
Irritability	66(27.16)	108(44.44)	69(28.40)	243(63.12)
Insomnia	57(23.65)	92(38.17)	92(38.17)	241(62.60)
Palpitations	100(45.45)	85(38.64)	35(15.91)	220(57.14)
Mental exhaustion	82(37.44)	117(53.42)	20(9.13)	219(56.88)
Unhappiness	96(50.0)	81(42.19)	15(7.81)	192(50.65)
Anxiety	89(53.29)	70(41.92)	8(4.79)	167(43.38)
Vaginal dryness	39(43.82)	32(35.96)	18(20.22)	89(23.12)
Urinary symptoms	12(40.0)	6(20.0)	12(40.0)	30(7.79)

In terms of subjective intensity of menopausal symptoms, loss of libido ranked top of the chart with 79.21% of the women classifying their symptom as severe. This was followed by urinary symptoms (40%), insomnia with 38.17% of the subjects indicating severe intensity and irritability expressed as severe by 28.40% respondents. For symptoms experienced with moderate intensity; tiredness and muscle pains ranked highest among the respondents with 78.96% and 70.33% of the women respectively. For the menopausal symptoms experienced with mild intensity; the most prevalent were anxiety and unhappiness in 53.29% and 50.0% respectively (Table 2).

**Figure 1:**
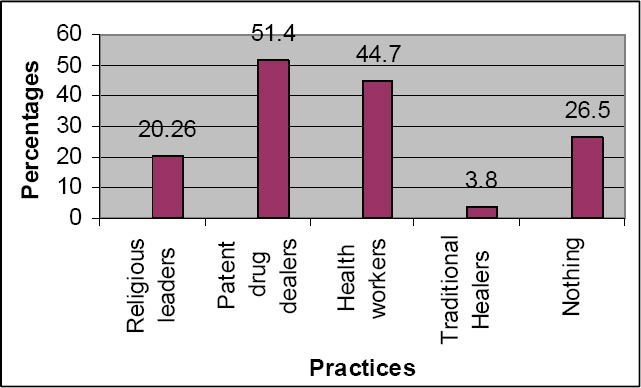
Health seeking practices of women with menopausal symptoms

Most of the patients (51.4%) consulted patent drug dealers followed by consultation with health workers (44.7%). It was only a small percentage (3.8%) of them that consulted the traditional healers (Figure 1).

## 4. Discussion

The most prevalent menopausal symptom reported by the participants was loss of libido, present in 356 (92.5%) of the women. This was followed by general body pain in 87.5%, waist pain 86.8% and joint pain in 85.5% of the subjects. Complaints of urinary symptoms were the least reported, present in only 30 (7.8%) women. Though similar symptoms were reported by other workers in Nigeria and elsewhere (Okonofua et al., 1990; Agwu, Umeora, & Ejikeme, 2008; Pan, Wu, Hsu, Yao, & Huang, 2002), the most prevalent symptoms vary between studies. Okonofua et al. (1990) in a study conducted in South-west Nigeria, found joint pain and hot flushes to be the most commonly reported menopause symptom. Agwu et al. (2008) in South East Nigeria, also found the commonest menopausal symptom to be hot flushes. It is interesting to note that, in the study by Agwu et al. (2008), urinary symptoms featured prominently, compared with the findings in this study where it was the least prevalent. The findings in this study are similar to the findings by Pan et al. (2002) among postmenopausal women in Taiwan. Among these Taiwanese women, insomnia was the most prevalent menopausal symptom reported in 42% of the subjects, followed by hot flushes in 38%. Furthermore, Friderichs (2005) in a survey of rural Xhosa postmenopausal women in South Africa, found that 80% of the subjects experienced hot flushes, with 20% describing them as extreme, while 69% experienced nocturnal sweating. In contrast to the pattern of menopausal symptoms described in the studies cited above, Aso (1998) found shoulder stiffness to be the commonest menopausal symptom in a study in the East Asian Region. Although Agwu et al. (2008) and Mahadeen et al. (2008) reported hot flushes as the commonest symptom, some Asian studies reported rather low prevalence of 12%-20% for hot flushes (Pan et al., 2002; Lock, 2001; Chim et al., 2002). Beyene and Martin (2001) in a study among Mayan women in Mexico reported to experience none of these symptoms.

Lumbago or low back pain was the most frequent menopausal symptom in Taiwan, and hand joints pains in Korea (WHO, 1981). These contradictory results may be explained by cultural, social, economic, psychic or physiological differences in patients in the different study locations (Punyahotra & Dennerstein, 1997). A great deal of the variance in reports of menopausal symptom prevalence, type, and severity may also be due to study design, including whether the study is cross-sectional or longitudinal, instruments used in the study and whether the study population is a community-based or clinical sample.

The high prevalence of loss of libido in this study could have emanated from the fact that most of the subjects in this study were widows and divorcees whose emotional status could have contributed to their experience. In addition, many cultures hold on to different beliefs about menstruation (Kumar & Srivastava, 2011). For example, in Nigeria, the Igbo society is rich in culture, myths and superstitions but, surprisingly no documentation exists on menstrual beliefs and practices among the population (Umeora & Egwuatu, 2008). Similar myths and superstition are found in the Niger Delta region of Nigeria where this study was conducted. One of such is the belief that menstruation cleanses a woman's system of impurities which predisposes to different disease conditions. Seminal fluid deposited in the vagina during menopause is believed to accumulate in the uterus and this predisposes to abdominal pain. The result is that a lot of women may lose interest in sexual activities during menopause to avoid the discomfort of abdominal pain and possibly enhance their longevity.

The widespread social inhibition and secrecy about female sexual and genital matters in most Nigerian communities may also be responsible for the low reporting of sexual and genital symptoms in previous studies (Nkwo, 2009). Women are much less embarrassed about reporting the non-genital and non-sexual complaints such as hot flushes, crawling sensation, insomnia, irritability and depression, hence the apparent higher prevalence of these non-genital and non-sexual symptoms. Information from focused group discussions however, revealed that Nigerian menopausal women experience the urinary and genital symptoms (Nkwo, 2009). These symptoms are usually presented to the clergy, spiritualists and traditional medicine men rather than to the hospitals (Agwu et al., 2008). Reddish (2002) therefore advised that researchers specifically ask about libido and associated symptoms. This was done during the interview and could have also accounted for the high prevalence of loss of libido among the subjects.

In this study, muscle pains, joint pains and tiredness were among the most prevalent menopausal symptoms the subjects presented with; however, it may be difficult to differentiate these from the symptoms of malaria which is endemic in this part of the continent. It is also possible that the high prevalence of joint pain and muscle pain may also be attributable to occupational hazards experienced by some of the subjects, 23.4% of who were farmers. The high incidence of musculoskeletal disorders with advancing age may contribute to the large number of participants presenting with muscle pain and joint pain.

Results on the severity of menopausal symptoms in this study showed that 28.25%, 49.84% and 21.9% were experiencing severe, moderate and mild menopausal symptoms respectively. This trend is similar to the findings on the severity of menopausal symptoms among Jordanian women in which 15.7%, 66.9% and 17.4% of them were experiencing severe, moderate and mild menopausal symptoms respectively (Gharaibeh, Al-Obeisat, & Hattab, 2010). The variation observed in the menopausal symptoms in different studies is also showing in the severity of symptoms. In this study, loss of libido ranked top of the chart in terms of severity followed by insomnia and waist pain. This is different from findings among the Jordanian women in which vasomotor signs were reported to have the highest scores for severity as manifested by hot flushes and night sweating.

Previous studies investigating factors influencing the prevalence, type, and severity of menopausal symptoms have found that lower educational level, lack of employment outside the home, and lower socioeconomic status are associated with increased prevalence and severity of menopausal symptoms (Dennerstein, Dudley, Hopper, Guthrie, & Burger, 2000; Polit & LaRocco, 1980). It is noteworthy that increased severity of menopausal symptoms is also associated with absence of a partner among other factors specifically in Korean postmenopausal women (Aso, 1998). Findings in this study are consistent with the prior studies.

The high prevalence of consultation of patent drug dealers in this study is similar to findings by Ige and Nwachukwu (2009) in South-Western Nigeria and Uzochukwu and Onwujekwe (2004) in South-Eastern Nigeria. Although their services are cheap and close to the people, this finding is a source of concern as they are often untrained and help to perpetrate the vicious circle of counterfeit drugs and death (Ige et al., 2009). The unfriendly attitude of health workers, perceived high cost of hospital services, lack of drugs and basic laboratory services, non-availability of a regular physician on site at the facility and delays in service in hospitals could have accounted for this (Sule et al., 2008). The women that did nothing about their symptoms could have done so because of the mild nature of the symptoms, financial constrain or poor attitude towards health related issues.

There are some limitations with the present study. First the women were asked to provide some retrospective information such as age of menopause hence the recall bias is unavoidable especially in the older women. Secondly, the data were collected without the verification of menopause status with blood hormone level testing. However, the use of standard symptom questions makes it feasible to compare our findings with other studies. The findings in this study cannot be applied to the general population since the women were recruited from primary care settings, and may have had more health problems, life stresses, and a higher incidence of psychological morbidity than women in the general population (Avis et al., 2001).

This study has implications for research, practice and education. Health-care providers especially at the primary care level need to play a more discernable and pragmatic role in constantly assessing the health needs of menopausal women as well as to implement appropriate health educational and promotional programs essential for improving the quality of life of menopausal women in Nigeria. Most of the menopause-associated symptoms studied were nonspecific and could be attributed to other medical and/or mental conditions or medications. Primary care providers must consider other factors/problems as well as menopause in their differential diagnosis. For example, night sweats in the environment of this study may be confused with the sensations of heat and sweating caused by hot weather. It has also been reported that night sweats experienced by primary care patients were associated with panic attacks, greater body mass index, chronic infection, sleep disturbances, antihistamines, and antidepressants, in addition to menopause (Mold, Mathew, Belgorem, & DeHaven, 2002). With the global increase in life expectancy, it is imperative to shift the focus of public health to address the emerging health issues of middle aged women. Strong emphasis needs to be laid to improve medical facilities to impart better services in accordance with changing need of women.

## 5. Conclusion and Recommendations

Loss of libido was the most prevalent menopausal symptom reported by the participants, followed by general body pain, waist pain and joint pain. Complaints of urinary symptoms were the least reported symptom. The observed menopausal symptoms are similar to findings in studies in different parts of the world but their prevalence and severity differ. These contradictory results could be explained by socio-cultural, economic, genetic, racial, physical and environmental differences in the different study locations. In spite of the available health facilities in these communities, the utilization of the services of patent drug dealers was very high. The utilization of the services of the traditional healers was poor.

There is an urgent need to intensify health education particularly about menopausal changes and encouraging women to have a more active lifestyle. They should be educated on the dangers of self-medication and patronage of patent drug dealers. Health-care providers need to consider treating these health issues from a lifestyle management perspective and precaution should be taken not to pathologize menopause.

Further studies on the impact of hormonal changes, diet, lifestyle and socio- cultural characteristics are also necessary to better understand menopausal experience.
